# Determinants of pentavalent and measles vaccination dropouts among children aged 12–23 months in The Gambia

**DOI:** 10.1186/s12889-022-12914-6

**Published:** 2022-03-17

**Authors:** Peter A. M. Ntenda, Alick Sixpence, Tisungane E. Mwenyenkulu, Kondwani Mmanga, Angeziwa C. Chirambo, Andy Bauleni, Owen Nkoka

**Affiliations:** 1 Malaria Alert Center (MAC), Kamuzu University of Health Sciences (KUHeS), Privative Bag 360, Chichiri, Blantyre, 3 Malawi; 2 Academy of Medical Sciences (AMS), Malawi University of Science and Technology (MUST), P.O Box 5196, Limbe, Malawi; 3 African Field Epidemiology Network, Ministry of Health, Expanded Programme on Immunization, P.O. Box 30377, Lilongwe, Malawi; 4 Malawi-Liverpool-Wellcome (MLW) Trust Clinical Research Programme, P.O. Box 30096, Mahtma Ghandi Road, Chichiri, Blantyre, Malawi; 5 Institute of Health & Wellbeing (IHW), University of Glasgow, 1st Floor 1055 Great Western Road, Glasgow, G12 0XH, Scotland, UK

**Keywords:** Immunization, Dropout, Coverage, Socioeconomic, Vaccination, Vaccine, Gambia

## Abstract

**Background:**

Every year, vaccination averts about 3 million deaths from vaccine-preventable diseases (VPDs). However, despite that immunization coverage is increasing globally, many children in developing countries are still dropping out of vaccination. Thus, the present study aimed to identify determinants of vaccination dropouts among children aged 12–23 months in The Gambia.

**Methods:**

The study utilized cross-sectional data obtained from the Gambia Demographic and Health Survey 2019–20 (GDHS). The percentage of children aged 12–23 months who dropped out from pentavalent and measles vaccination were calculated by (1) subtracting the third dose of pentavalent vaccine from the first dose of Pentavalent vaccine, and (2) subtracting the first dose of measles vaccine from the first dose Pentavalent vaccine. Generalized Estimating Equation models (GEE) were constructed to examine the risk factors of pentavalent and measles vaccinations dropout.

**Results:**

Approximately 7.0% and 4.0% of the 1,302 children aged 12–23 months had dropped out of measles and pentavalent vaccination respectively. The multivariate analyses showed that when caregivers attended fewer than four antenatal care sessions, when children had no health card or whose card was lost, and resided in urban areas increased the odds of pentavalent dropout. On the other hand, when women gave birth in home and other places, when children had no health card, and being an urban areas dweller increased the odds of measles dropout.

**Conclusion:**

Tailored public health interventions towards urban residence and health education for all women during ANC are hereby recommended.

## Background

Vaccination is considered the most successful and cost-effective public health intervention against infectious diseases [1, 2]. Each year, about 3 million deaths among children are averted from vaccine-preventable diseases (VPDs) such as diphtheria, tetanus, pertussis, influenza, and measles [3]. However, by the end of 2020, the global coverage of childhood vaccination dropped from 86% in 2019 to 83% [4]. It is reported that the coronavirus disease 2019 (COVID-19) pandemic and its associated disruptions have strained health systems as a result, about 23 million under one children did not receive basic vaccines in 2020 [4]. In The Gambia, the coverage of individual immunization is high (90% or above) except for oral polio vaccine (OPV) 4 and complete immunization which were reported at 85% each in 2020 respectively [5]. It is known that the high coverages are due to high public awareness, with the accessibility of vaccination services through permanent outreach sites for remote areas and static reproductive and child health (RCH) clinics [5]. Regarding the multi-dose vaccines, the coverage is reported to be the highest for the first dose and falls in subsequent doses. Precisely, the coverage rates for the initial dose of diphtheria, pertussis, and tetanus (DPT), pneumococcal conjugate vaccine (PCV), and rotavirus vaccine (RV) were reported at 98%, 99%, and 98%, respectively. Nevertheless, the coverages for the last dose of each antigen dropped to 93%, 92%, and 95%, respectively [6].

Immunization dropout signifies that the child has received the first recommended dose of the vaccine and yet has missed the next recommended dose [7]. Studies have reported on the various characteristics that influence childhood immunization dropout [7–10]. For example, in Ethiopia, it was reported that counseling for mothers about vaccination; fear of vaccine side effects; postnatal care (PNC) attendance, and having a mother who did not receive tetanus toxoid (TT) vaccination were independent factors of vaccination dropout [7]. In Nepal, mothers with less than 4 or no antenatal care (ANC) visits, long distances to the health facilities, province, and mother without formal employment were reported to be factors associated with vaccination dropout [8]. Furthermore, in Kenya, having a caregiver with below secondary education and residing >5 km from the health facilities were associated with higher odds of dropping out. On the other hand, caregivers who received reminder text messages were less likely to drop out [9]. Elsewhere in Ghana, children who had no immunization cards were more likely to drop out compared to those who possessed it [10]. And finally, in urban Pakistan, in a randomized controlled trial, it was reported that a significant increase in DPT3 completion was estimated in the group that received both redesigned card and center-based education compared with the standard care group [11].

Over the years, immunization coverage in The Gambia has improved such that the proportion of children aged 12-23 months who received all basic vaccinations increased from 76% in 2013 to 85% in 2019-20 [6]. However, this coverage is still trailing behind the target that was set by the Global Vaccine Action Plan (GVAP) of 90% coverage for all antigens at the national level and 80% coverage for all antigens at the districts level by 2020 [12]. These statistics may indicate that a certain proportion of children are dropping out of immunization programs. It is noted that a few researchers have focused on the factors associated with either individual immunization coverage [13], full immunization [13, 14], or non-vaccination in the Gambia [2, 15]. However, only one study reported the dropouts between vaccine doses [16], yet no single associated factor was considered in that study. Therefore, the aim of the present study was to identify the determinants of immunization dropouts among Gambian children aged 12–23 months. The findings of this study will help the program designers to improve the EPI program performance in The Gambia.

## Methods

### Data source, design, and sampling methods

This study used data obtained from the 2019-20 Gambia Demographic and Health Survey (GDHS) [6]. The GDHS used a cross-section study design and was carried out by The Gambia Bureau of Statistics (GBoS) in conjunction with the Gambia Ministry of Health (MoH) [6]. The GDHS was designed to yield a nationally representative sample using two-stage cluster sampling technique. Enumeration areas (EAs) were selected with a probability proportional to their size within each sampling stratum in the first stage and yielded 281 EAs [6]. In the second stage, the households were systematically sampled from the EAs using a household listing [6]. Thus, the resulting lists of households served as the sampling frame from which a fixed number of 25 households were systematically selected per cluster.

### Setting and immunization services in The Gambia

The Expanded Programme on Immunisation (EPI) in The Gambia officially started in May 1979 where six vaccines were recommended [13]. The ultimate goal was to administer Bacillus Calmette–Guérin (BCG) vaccine against tuberculosis (TB), oral polio vaccine (OPV) against poliomyelitis, DPT vaccine against diphtheria, tetanus and pertussis, and yellow fever vaccine to protect against yellow fever [13, 15]. Over the last two decades, the programme has been introducing new and underused vaccines into The Gambian routine services. These vaccines include hepatitis B – HepB (introduced in 1990), *Haemophilus influenzae* type b – Hib (introduced in 1997), pneumococcal conjugate vaccine – PCV (introduced in 2009), measles-mumps-rubella second dose – MMR (2012), rotavirus vaccine – RV (introduced in 2013), inactivated polio vaccine – IPV (introduced in 2015), meningitis A – MenA (introduced in 2019), and human papillomavirus vaccine – HPV (introduced in 2019) [2, 15]. The Gambia routine immunization programme recommends that the BCG, the first dose of polio, first dose of HepB should be given at birth [13]. Further, it is recommended that the DTP/Hib/HepB combined (Pentavalent vaccine); the second, the third, the fourth dose of polio; PCV and RV should be given at approximately 2, 3 and 4 months respectively [13]. The measles, yellow fever, fourth dose of polio are recommended to be administered as soon as the child reaches 9 months of age [13]. While the DTP/Hib/HepB combined and fifth dose polio should be administered at 18 months respectively [13]. Lastly, vitamin A should be provided every 6 months (from 6 months of age till the child is 59 months) [13]. Table 1 shows the schedule of the Gambian childhood expanded programme on immunization.

**Table 1:** The Schedule of the Gambian Childhood Expanded Programme on Immunization

Schedule	Vaccinations
At birth	BCG vaccine, *OPV0 vaccine, first dose of HepB vaccine
2 months	First dose of Pentavalent; OPV, PCV, and RV
3 months	Second dose of Pentavalent; OPV, PCV, and RV
4 months	Third dose of Pentavalent; OPV, and PCV
9 months	First dose of MR vaccine; yellow fever vaccine, fourth dose of OPV
18 months	Fourth dose of Pentavalent (booster); Firth dose OPV; second dose of MR
Other	Vitamin A every 6 months (from 6 months of age till 59 months)

*Usually is given shortly after birth; *BCG* Bacillus Calmette–Guérin, Pentavalent (DTP, Hib, HBV), where *DTP* diphtheria, tetanus, pertussis, *HepB* hepatitis B virus, *Hib* *Haemophilus influenzae* type b, *OPV* Oral Poliovirus Vaccine, *PCV *pneumococcal conjugate vaccine, *RV* Rotavirus vaccine, *MR* Measles-Rubella vaccine

### Data collection

All women aged 15-49 years who were either permanent residents of the selected households or visitors who stayed in the households the night before the survey were eligible to be interviewed. Data were collected using face-to-face interviews on the measures of population health, including maternal and child health indicators [6].

### Inclusion and exclusion criteria

**Fig. 1:**
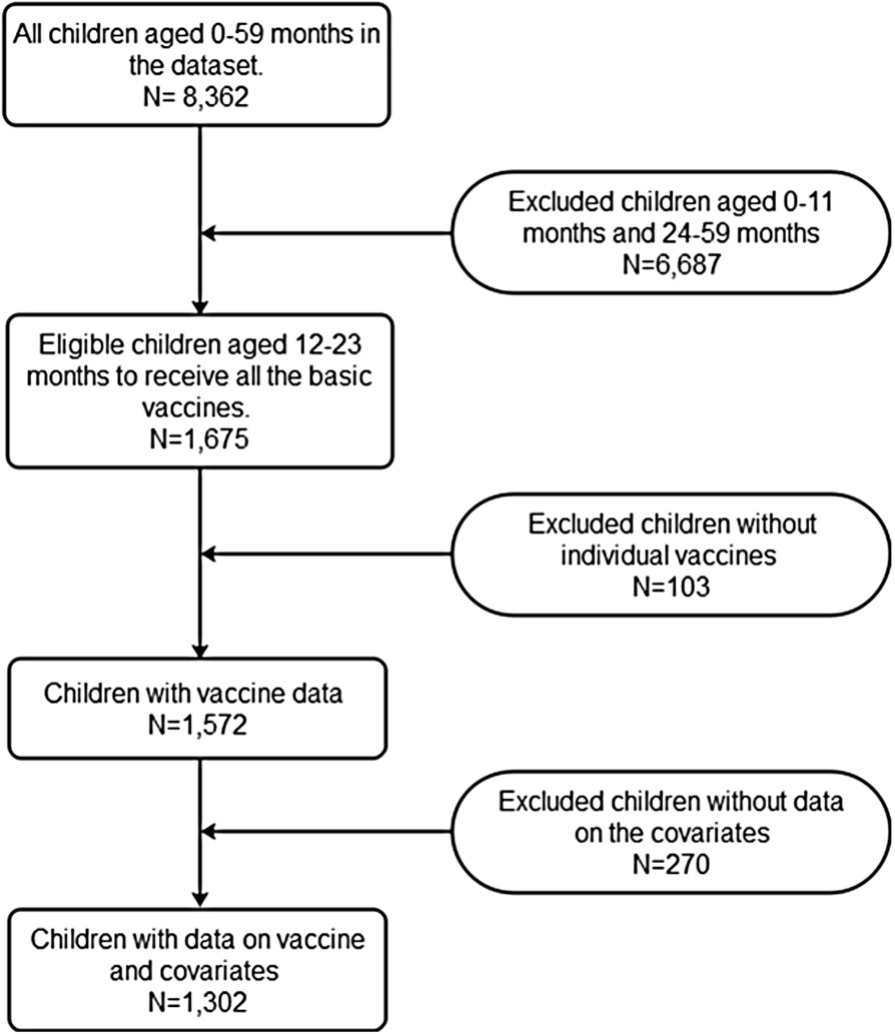
Inclusion and exclusion criteria.

The analysis was limited to children of age group 12 to 23 months because children of this age group are expected to have completed all the basic vaccines. However, all children who did not receive an individual vaccine were excluded from the analysis. Furthermore, all children who had missing data on the other covariates were excluded from this study. Figure 1 shows the inclusion and exclusion criteria.

### Dependent variable

The dependent variable of the current study was immunization dropout and it indicates that one has received the first recommended dose of vaccine and missed the next recommended dose [8]. In this study, dropout was defined as the child who received the first antigen of pentavalent but not the third antigen of the Pentavalent or the first antigen of pentavalent but not first antigen of measles [12]. Furthermore, the Pentavalent dropout rate was calculated by dividing the number of children aged 12–23 months who received pentavalent1 minus the number of children aged 12–23 months who received pentavalent3 divided by the number of children 12–23 months of age who received Pentavalent1 multiplied by 100 (Pentavalent1–Pentavalent3) ÷ Pentavalent1 x 100%) [12]. It was also calculated as the percentage of children aged 12–23 months who received pentavalent1 and measles1 divided by those who received pentavalent1 multiplied by 100 (Pentavalent1–measles) ÷ Pentavalent1 x 100%) [12]. The World Health Organization (WHO) recommends that the dropout rates of both the Penta1 to Penta3 and Penta1 to MCV1 should be <10% [12]. It should be noted that a dropout rate of >10% reflects underutilization of immunization services.

### Independent variables

The following characteristics were considered as predictors of immunization dropout after a review of relevant literature [8, 17]. Sex of the child (male and female), the birth order (1, 2–3, 4–5, and 6 and above), place of delivery (health facilities or homes and other places), mother’s age (15–24, 25–34, and ≥35 years), the mother’s and husband’s education (no formal education, primary school education, and secondary and higher education), ANC visits (adequate or inadequate visits), immunization card (no card and had the card but its whereabouts was unknown and had the card and its whereabouts was known), the household wealth index (poorest, poorer, middle, richest, and richest), tetanus toxoid injection during pregnancy (received or not received), number of children under the age of 5 years (0–1, 2, 3 or more), distance to the health facility (big problem, not a big problem), amount of media exposure (0, 2, and 3), the place of residence (urban or rural), and local government area (Banjul, Kanifing, Brikama, Mansakonko, Kerewan, Kuntaur, Janjanbureh, and Basse). The household wealth index was generated through a principal component analysis using information easy-to-collect data on a household’s ownership of selected assets, such as televisions and bicycles [18].

### Statistical analysis

All analyses were conducted separately for pentavalent and measles dropout outcomes. Descriptive analyses were performed to describe the baseline characteristics of the study population. Univariate and multivariate analyses were conducted using generalized estimating equations (GEE) for estimating the effects of predictors on the risk of childhood immunization dropouts. Since children residing in the same household, communities, and belonging to the same mother may be more similar to each other, GEE models were used to adjust for the clustering within the household and communities. The results of the multivariate analysis were obtained using adjusted odds ratios (aORs) with their *P*-values and 95% confidence intervals (CIs). SAS software version 9.4 (SAS Institute Inc., Cary, NC, USA) was used to conduct all of the analyses.

### Ethical considerations

All methods were carried out in accordance with the Declaration of Helsinki. The 2019-2020 GDHS was implemented by The GBos in conjunction with the Gambia Ministry of Health. The protocols and procedures for GDHS were reviewed and approved by The Gambian Government/Medical Research Council Joint Ethics Committee and the Institutional Review Board (IRB) of ICF Macro. ICF IRB ensures that the survey complies with the U.S. Department of Health and Human Services regulations for the protection of human subjects (45 CFR 46), while the host country IRB ensures that the survey complies with laws and norms of the nation [19]. During survey implementation, informed consent was sought from participants prior to each interview and a parent or guardian provided consent prior to participation by children less than 18 years. The authors obtained permission from the DHS program for the use of the data beyond the primary purpose of the survey.

## Results

### Baseline characteristics of the study sample and dropouts rates

**Fig. 2:**
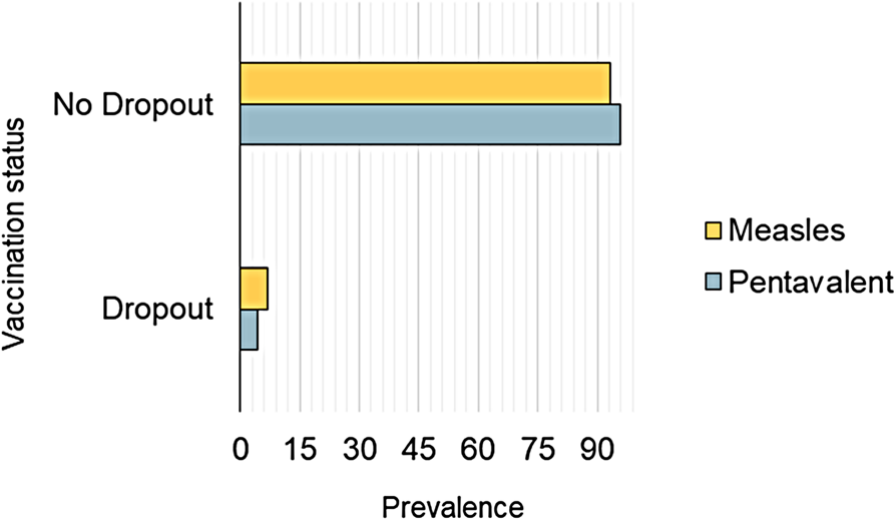
Percentage of children with Pentavalent 3 and Measles 1 dropouts.

Overall, 1.302 children aged 12–23 months were analyzed in this study. The dropouts for the measles vaccine and the Pentavalent vaccine were reported at 6.8% and 4.3% respectively (Fig. 2 shows the vaccination dropouts). Table 2 presents the baseline characteristics of the study population. More than half of the children (52%) were male and one-third (34.3%) of the children were in the 2–3 birth order. As regards maternal and household characteristics, more than half (54.5%) of respondents were distributed in the age group 25–34 years and 52.2% of respondents had primary school education. Furthermore, about two-thirds (63%) of respondents their husbands had no formal education. A majority of respondents (59.4%) had more than three under-5-year-old children, and a half (47%) of the respondents had access to at least two types of mass media. In terms of health service utilization, a majority (81%) of births occurred in health faculties, 95% had an immunization card, 82% had adequate antenatal visits, and 88% had tetanus toxoid injection during pregnancy. Nearly 31% of respondents had big problems with distance to the nearest health facility. In terms of community characteristics, a majority of respondents were rural (58%).

**Table 2:** Baseline characteristics of the study population in the Gambian DHS (*N*=1,302)

Characteristic	n	*%*	95% CL^a^	
Sex				
Male	681	52.3	49.6, 55.0	
Female	621	47.7	45.0, 50.4	
Birth order				
1	228	17.5	15.4, 19.6	
2–3	446	34.3	31.7, 36.8	
4–5	330	25.3	23.0, 27.7	
6+	298	22.9	20.6, 25.2	
Mother’s age (years)				
15–24	292	22.4	20.2, 24.7	
25–34	710	54.5	51.8, 57.2	
35–49	300	23.0	20.7, 25.3	
Mother’s education				
No formal education	680	52.2	49.5, 54.9	
Primary education	251	19.3	17.1, 21.4	
Secondary and higher	371	28.5	26.0, 30.9	
Husband’s education				
No formal education	816	62.7	60.0, 65.3	
Primary education	82	6.3	5.0, 7.6	
Secondary and higher	404	31.0	28.5, 33.5	
Place of delivery				
Home or other	249	19.1	17.0, 21.3	
Health facility	1053	80.9	78.7, 83.0	
Number of antenatal visits				
<4	229	17.6	15.5, 19.7	
4+	1073	82.4	80.3, 84.5	
Possession of immunization card				
No longer have a card	35	2.7	1.8, 3.6	
Had card and its whereabouts known	1240	95.2	94.1., 96.4	
Had a card but its whereabouts unknown	27	2.1	1.3, 2.8	
TTI during pregnancy				
Not received	154	11.8	10.1, 13.6	
Received	1148	88.2	86.4, 89.9	
No of under-5-year children				
0–1	191	14.7	12.7, 16.6	
2	338	26.0	23.6, 28.3	
3+	773	59.4	56.7, 62.0	
Household wealth				
Poorest	476	36.6	33.9, 39.2	
Poorer	279	21.4	19.2, 23.7	
Middle	270	20.7	18.5, 22.9	
Richer	164	12.5	10.8, 14.4	
Richest	113	8.7	7.1, 10.2	
Amount of media exposure				
0	153	11.8	10.0, 13.5	
1	434	33.3	30.8, 35.9	
2	658	50.5	47.8, 53.3	
3	57	4.4	3.3, 5.5	
Distance to the health facility				
Not a big problem	903	69.4	66.8, 71.9	
Big problem	399	30.6	28.1, 33.2	
Place of residence				
Urban	547	42.0	39.3, 44.7	
Rural	755	58.0	55.3, 60.7	
Local government area				
Banjul	61	4.7	3.5, 5.8	
Kanifing	103	7.9	6.4, 9.4	
Brikama	205	15.7	13.8, 17.7	
Mansakonko	125	9.6	8.0, 11.2	
Kerewan	181	13.9	12.0, 15.8	
Kuntaur	200	15.4	13.4, 17.3	
Janjanbureh	163	12.5	10.7, 14.1	
Basse	264	20.7	18.1, 22.5	

^a^*CL* Confidence Limits for Percent, *TTI* Tetanus Toxoid Injection

### Factors associated with pentavalent vaccine dropout

Table 3 displays univariate and multivariate logistic regression results of pentavalent vaccination dropout. Compared to children whose caregivers had adequate ANC visits, the odds of experiencing pentavalent dropout (aOR: 2.44; 95% CI: 1.16–5.12) were high among children whose caregivers had inadequate ANC visits. Furthermore, the odds of experiencing pentavalent vaccination dropout was much higher among children whose caregivers who had no vaccination card/no longer had a card (aOR: 12.4; 95% CI: 4.09–37.8) and who had a card but its whereabouts were not known (aOR: 32.7; 95% CI: 10.7–100.0) compared with children who had vaccination card and its whereabouts was known. Additionally, the odds of experiencing pentavalent vaccination dropout were significantly higher among children from the urban areas (aOR: 9.30; 95% CI: 2.80–30.9), compared to children from rural areas.

**Table 3:** Factors associated with Pentavalent 3 and Measles 1 vaccination dropouts

Vaccine		Pentavalent dropout				Measles dropout			
		OR 95% (CI)	*P*-value	aOR 95% (CI)	*P*-value	OR 95% (CI)	*P-*value	aOR 95% (CI)	*P-*value
Sex of the child									
	Male	1.84 (0.97–3.51)	0.0618	1.97 (0.99–3.91)	0.0544	1.05 (0.66–1.66)	0.8387	1.08 (0.68–1.72)	0.7389
	Female	1.00		1.00		1.00		1.00	
Birth order									
	1	1.31 (0.49–3.50)	0.5841	1.11 (0.23–5.40)	0.8995	1.62 (0.80–3.29)	0.1777	1.20 (0.42–3.43)	0.7386
	2–3	1.26 (0.53–3.01)	0.6034	1.02 (0.29–3.54)	0.9763	0.98 (0.51–1.91)	0.9588	0.74 (0.31–1.75)	0.4864
	4–5	1.23 (0.49–3.11)	0.6571	1.37 (0.44–4.30)	0.5896	1.28 (0.65–2.53)	0.4785	1.16 (0.54–2.52)	0.7068
	6+	1.00		1.00				1.00	
Mother’s age (years)									
	15–24	0.93 (0.38–2.31)	0.8763	0.87 (0.21–3.64)	0.8520	1.25 (0.64–2.43)	0.5176	1.21 (0.46–3.22)	0.6989
	25–34	0.96 (0.45–2.07)	0.9232	0.98 (0.35–2.75)	0.9643	0.98 (0.55–1.76)	0.9498	1.01 (0.50–2.07)	0.9717
	35–49	1.00		1.00		1.00		1.00	
Mother’s education									
	No formal education	0.79 (0.39–1.60)	0.5107	0.54 (0.22–1.31)	0.1726	0.99 (0.57–1.72)	0.9602	1.00 (0.53–1.90)	0.9903
	Primary education	0.77 (0.32–1.89)	0.5727	0.62 (0.22–1.77)	0.3710	1.19 (0.62–2.29)	0.6028	1.27 (0.62–2.57)	0.5134
	Secondary and higher	1.00		1.00		1.00		1.00	
Husband’s education									
	No formal education	1.66 (0.78–3.55)	0.1878	2.70 (0.98–7.44)	0.0545	1.32 (0.76–2.27)	0.3242	1.79 (0.94–3.41)	0.0746
	Primary education	2.22 (0.68–7.28)	0.1869	3.02 (0.75–12.1)	0.1201	1.93 (0.78–4.79)	0.1576	2.32 (0.90–6.02)	0.0825
	Secondary and higher	1.00		1.00		1.00		1.00	
Place of delivery									
	Home or other	1.07 (0.49–2.32)	0.8708	1.46 (0.62–3.43)	0.3823	1.36 (0.77–2.41)	0.2894	1.86 (1.02–3.40)	0.0429
	Health facility	1.00		1.00		1.00		1.00	
Number of antenatal visits									
	<4	2.77 (1.40–5.45)	0.0034	2.44 (1.16–5.12)	0.0184	1.54 (0.89–2.70)	0.1240	1.46 (0.83–2.58)	0.1944
	4+	1.00		1.00		1.00		1.00	
Immunization card									
	No longer have a card	12.7 (3.95–40.0)	<0.0001	12.4 (4.09–37.8)	<0.0001	4.97 (1.79–12.7)	0.0020	3.99 (1.42–11.2)	0.0086
	Its whereabouts unknown	44.6 (12.1–163.9)	<0.0001	32.7 (10.7–100.0)	<0.0001	2.01 (0.52–7.72)	0.3111	1.79 (0.46–7.04)	0.4037
	Its whereabouts known	1.00		1.00		1.00		1.00	
TTI during pregnancy									
	Not received	0.87 (0.32–2.34)	0.7764	0.68 (0.22–2.09)	0.5016	0.73 (0.33–1.58)	0.4123	0.55 (0.24–1.27)	0.1625
	Received	1.00		1.00		1.00		1.00	
No of under-5 children									
	0–1	3.48 (1.63–7.43)	0.0013	1.72 (0.70–4.22)	0.2368	2.23 (1.25–3.96)	0.0064	1.64 (0.86–3.12)	0.1349
	2	1.29 (0.60–2.78)	0.5162	1.04 (0.50–2.41)	0.9267	0.91 (0.50–1.64)	0.7399	0.80 (0.43–1.48)	0.4726
	3+	1.00		1.00		1.00		1.00	
Household wealth									
	Poorest	1.74 (0.41–7.23)	0.4573	4.10 (0.65–26.0)	0.1341	0.41 (0.18–0.94)	0.0352	1.01 (0.31–3.36)	0.9852
	Poorer	1.79 (0.41–7.82)	0.4364	2.24 (0.45–11.2)	0.3251	0.61 (0.26–1.42)	0.2524	0.79 (0.29–2.14)	0.6426
	Middle	1.78 (0.42–7.50)	0.4348	0.97 (0.21–4.47)	0.9735	0.61 (0.26–1.41)	0.2477	0.45 (0.18–1.14)	0.0912
	Richer	2.21 (0.50–9.82)	0.2978	1.08 (0.23–5.07)	0.9235	0.80 (0.33–1.92)	0.6137	0.63 (0.25–1.57)	0.3209
	Richest	1.00		1.00		1.00		1.00	
Amount of media exposure									
	0	0.89 (0.14–5.66)	0.9029	0.88 (0.10–7.51)	0.9072	0.74 (0.17–3.33)	0.6970	0.85 (0.17–4.14)	0.8353
	1	1.41 (0.29–6.91)	0.6696	1.56 (0.24–10.4)	0.6433	1.54 (0.42–5.56)	0.5129	1.68 (0.43–6.52)	0.4527
	2	1.00 (0.21–4.78)	0.9989	0.86 (0.14–5.51)	0.8766	1.33 (0.38–4.72)	0.6556	1.36 (0.37–5.06)	0.6461
	3	1.00		1.00		1.00		1.00	
Distance to the health facility									
	Big problem	1.64 (0.77–3.50)	0.2038	1.33 (0.58–3.04)	0.4988	1.54 (0.87–2.74)	0.1367	1.22 (0.67–2.22)	0.5134
	Not a big problem	1.00		1.00		1.00		1.00	
Place of residence									
	Urban	3.15 (1.54–6.47)	0.0018	9.30 (2.80–30.9)	0.0003	3.62 (2.15–6.09)	<0.0001	6.24 (2.69–14.5)	<0.0001
	Rural	1.00		1.00		1.00		1.00	
Local government area									
	Banjul	2.68 (0.63–11.3)	0.1799	1.27 (0.29–5.62)	0.7474	2.33 (0.79–6.80)	0.1241	1.02 (0.35–2.94)	0.9721
	Kanifing	2.67 (0.75–9.38)	0.1289	1.49 (0.39–5.63)	0.5601	1.12 (0.39–3.20)	0.8361	0.52 (0.18–1.48)	0.2200
	Brikama	1.56 (0.47–5.12)	0.4668	0.64 (0.19–2.16)	0.4730	1.56 (0.66–3.68)	0.3106	0.85 (0.37–1.94)	0.6941
	Mansakonko	0.58 (0.10–3.31)	0.5362	0.53 (0.09–3.17)	0.4881	0.41 (0.11–1.50)	0.1760	0.56 (0.16–1.97)	0.3638
	Kerewan	0.20 (0.02–1.78)	0.1473	0.19 (0.02–1.80)	0.1466	0.75 (0.27–2.06)	0.5764	1.09 (0.44–2.73)	0.8509
	Kuntaur	1.09 (0.29–4.06)	0.8938	1.04 (0.28–3.90)	0.9586	0.85 (0.32–2.26)	0.7421	1.34 (0.54–3.37)	0.5286
	Janjanbureh	3.31 (1.02–10.8)	0.0472	3.17 (0.97–10.3)	0.0559	0.70 (0.24–2.01	0.5014	0.86 (0.32–2.30)	0.7554
	Basse	1.00		1.00		1.00		1.00	

### Factors associated with measles vaccine dropout

Table 3 shows also the univariate and multivariate logistic regression results of measles vaccination dropout. Compared to children whose caregivers had given birth in health facility, the odds of experiencing measles dropout (aOR: 1.86; 95% CI: 1.02–3.40) were high among children whose caregivers whose deliveries occurred in homes or other places. Furthermore, the odds of experiencing measles vaccination dropout was much higher among children whose caregivers who had no vaccination card/no longer had a card (aOR: 3.99; 95% CI: 1.42–11.2) compared with children who had vaccination card and its whereabouts were known. Additionally, the odds of experiencing measles vaccination dropout were significantly higher among children from the urban areas (aOR: 6.24; 95% CI: 2.69–14.5), compared to children from rural areas.

## Discussion

The current study aimed to identify determinants of vaccination dropouts among children aged 12–23 months in The Gambia. The initial against subsequent doses of pentavalent vaccine (usually third) is regarded as a tracer indicator. Routinely, dropout is used as an indicator of immunization program performance and low dropout rates indicate good access and utilization of immunization services [20]. Generally, if an infant defaults to the three doses of pentavalent vaccine, it specifies that there is an access problem while a high dropout rate between Penta1 and the measles immunization suggests a service utilizations problem [21]. Further, the MCV dropout rate assesses whether the program is able to vaccinate children beyond the first year of life [20]. The World Health Organization (WHO) recommended that DTP1 to DTP3, BCG to measle-containing virus (MCV1), and MCV1 to MCV2 should be used as the indicators of immunization dropout [20]. The WHO emphasizes that if the dropout rate is more than 10%, then it indicates that many people are not using the services [12].

The present study reported that the dropout rates for measles and pentavalent vaccines were 6.8% and 4.3% respectively. These results are somewhat lower than those reported in a previous study [16] that used data obtained from the 2013 survey and below the 10% cut-off recommended by WHO [22], thus indicating an improvement in immunization coverage in The Gambia. It is reported that the recent gains in immunization coverage are due to the support from The Global Alliance for Vaccines and Immunizations (Gavi), the Vaccine Alliance which work with the Civil Society Organizations (CSOs), Non-Governmental Organizations (NGOs), WHO, and other United Nation (UN) agencies to support the Government of The Gambia by ensuring that all children receive all their basic vaccinations [23]. Furthermore, Vaccine Alliance also supports the Gambian government in the procurement and management of all vaccines and cold chain equipment, to ensure a constant supply of vaccines and equipment needed to transport and store vaccines at all levels. This includes constructing storage rooms and equipping facilities with solar-powered cold chain equipment to ensure all vaccines reach all children without losing their potency. Additionally, the vaccine alliance exerts its efforts to increase access to immunization services, through the extension of service delivery points in areas of low coverage attributed to access [23].

In line with previous literature on childhood immunization in general [24–26], having less than 4 ANC visits was significantly associated with an increased risk of having pentavalent vaccination dropout. The previous studies have hypothesized, caregivers who underutilize ANC services do not have the chance to receive information about the benefit and schedule of vaccination [27]. Furthermore, another probable reason for the Pentavalent vaccine dropout maybe that caregivers who default ANC services nor gave birth in health facilities may place little or no value of childhood immunization than their counterparts of the same socioeconomic background and that they may miss out on counseling about child immunization in the postnatal period [28].

Consistent with prior research on immunization coverage [3, 29, 30], the current study found that women who gave birth at home and other places had increased chances of experiencing measles vaccination dropout. For instance, a study on the impact of maternal health care utilization on routine immunization coverage of children in Nigeria found that ANC attendance irrespective of the number of visits had positive effects on the child being fully immunized after adjusting for covariates [31]. Additionally, in Ethiopia [29] it was reported that delivery at health facilities was significantly associated factors with full immunization, likely because some vaccines, such as BCG and OPV 0 are habitually given immediately after birth at the health facilities. Moreover, mothers who gave birth at the health facilities are probably more health-conscious and thus more likely to have their children adhere to the vaccination services.

Consistent with results from prior studies on immunization coverages and immunization dropout [10, 24, 32, 33], the current study found that children who had no card or had the card but it was displaced were more likely to experience both pentavalent and measles dropouts. Generally, an immunization card is a paper-based platform that is used to record and track immunization coverage [34]. Prior studies demonstrated that caregivers with child health cards could easily follow the immunization schedule and thus can be able to attain timely immunization for their children [24, 35]. Moreover, having a well-kept immunization card with a clearly-labeled schedule can well remind caregivers about timely childhood immunization [24, 36]. Researchers in Ghana hypothesized that caregivers may default subsequent vaccination schedule due to ill-treatment they could experience from health care providers when they are informed of the lack of child immunization card i.e. owing to misplacement, loss or spoiled [37]. Furthermore, elsewhere it was reported that lack of immunization card may mean that some antigens may have been administered to the children but because there are absent records, caregivers could easily forget that no immunizations were given.

The current study also found that immunization dropout varied by area of residence. Specifically, children in the urban settings were more likely to have the Pentavalent and measles immunization dropouts. Many studies on rural-urban inequities in immunization have placed rural children to be at disadvantage both in the proportion receiving full immunization and individual vaccines [38]. However, other studies have reported that children in rural areas are more likely to complete the required vaccinations [39, 40]. The reasons why in some settings children in urban areas have high vaccine coverage and less dropout rate may be that; 1) caregivers may be highly educated thus may have increased autonomy, changes in traditional beliefs, and control over household resources [41]. In turn, they may have an enhanced healthcare-seeking behavior and may be able to comprehend new health knowledge more quickly [24], and 2) caregivers may dwell in richer households, thus, they might not have barriers to access services at the health facilities compared to poor families [42]. Nonetheless, the findings of the current study are in line with previous studies in other developing countries [43, 44] where immunization coverage was higher in rural areas than urban townships. One reason that explains high immunization coverages in rural areas is the use of the traditional birth attendants (TBA) and primary health care (PHC) workers that both play a role in encouraging mothers to attend the maternal and child health (MCH) clinics of which these roles do not formally exist in urban areas [13]. Another possible reason why immunization coverages are high and dropouts are low in rural areas might be due to the establishment and use of outreach clinics. It is established that sustained outreach is an approach for reaching remote areas of the population with limited access to immunization locations [45]. Outreach clinics encourage health care workers to take vaccines from fixed health facilities and travel to remote locations to immunize children thus minimizing the chances of caregivers defaulting immunization services [46]. Many low-and-middle-income countries (LMICs), supplement community health volunteers (CHVs) in various essential health services. Indeed, it is reported that CHVs could help improve access to and use of essential health services such as immunization by communities in LMICs [47]. One of the responsibilities of the CHVs is to regularly visit families in their homes to provide counselling about reproductive, maternal, newborn and child health (RMNCAH) and other health concerns [48].

### Strengths and limitations

The inferences drawn in this study could be generalized to all children aged 12-23 months in The Gambia owing to the use of a nationally representative sample. However, these results should be interpreted with caution: Firstly, the current study utilized a cross-sectional study design, thus causal and temporal inferences cannot be drawn. Second, information on immunization was collected from vaccination cards, thus the findings of this study are prone to recall bias, as the respondents who did not have health cards were asked to recall vaccines a child had received. Thirdly, the datasets used in this study did not report any vaccine stockouts, accessibility of immunization services, and inconsistent scheduling of vaccination supply.

## Conclusion

Tailored public health interventions towards the urban residence and health education for all mothers attending maternal and child health services (such as ANC and PNC) on child vaccination completion are hereby recommended. Furthermore, since children without health passports or health profiles had increased chances of dropping out from immunization, it is, therefore, necessary to develop an android based system with automatic reminder functionalities sent to the health workers and wherever possible to the guardians about the next schedule for all children due for vaccination in order to reduce the risk of defaulting immunization services.

## Data Availability

The datasets generated and/or analyzed during the present study are available in The DHS Program repository, https://dhsprogram.com/data/available-datasets.cfm

## Abbreviations

AIDS: Acquired Immunodeficiency Syndrome
ANC: Antenatal Care
AORs: adjusted Odds Ratios
BCG: Bacillus Calmette–Guérin
CIs: Confidence Intervals
CHVs: Community Health Volunteers
COVID-19: Coronavirus disease 2019
DPT: Diphtheria, Pertussis, and Tetanus
EAs: Enumeration Areas
EPI: Expanded Programme on Immunisation
GBoS: Gambia Bureau of Statistics
GDHS: Gambia Demographic and Health Survey
GEE: Generalized Estimating Equations
GVAP: Global Vaccine Action Plan
HepB: hepatitis B
Hib: Haemophilus influenzae type b
HIV: Human Immunodeficiency Virus
HPV: Human Papillomavirus Vaccine
LMICs: Low-and-Middle-Income-Countries
MCV: Measles Containing Virus
MenA: Meningitis A
MoH: Ministry of Health
NC: New York City
OPV: Oral Polio Vaccine
PCV: Pneumococcal Conjugate Vaccine
PHC: Primary Health Care
PNC: Post-natal Care
RCH: Reproductive and Child Health
RMNCAH: Maternal, New Born and Child Health
RV: Rotavirus Vaccine
STIs: Sexually Transmitted Disease
TB: Tuberculosis
TBA: Traditional Birth Attendants
USA: United States of America
VPDs: Vaccine Preventable Diseases
WHO: World Health Organization
